# The relationships between body composition characteristics and cognitive functioning in a population-based sample of older British men

**DOI:** 10.1186/s12877-015-0169-y

**Published:** 2015-12-21

**Authors:** Efstathios Papachristou, Sheena E. Ramsay, Lucy T. Lennon, Olia Papacosta, Steve Iliffe, Peter H. Whincup, S. Goya Wannamethee

**Affiliations:** Department of Primary Care & Population Health, UCL Medical School, Royal Free Campus, Rowland Hill Street, London, NW3 2PF, UK; Population Health Research Institute, St George’s University of London, Cranmer Terrace, London, SW17 0RE, UK

**Keywords:** Obesity, Adiposity, Sarcopenia, Cognition, Dementia, Ageing

## Abstract

**Background:**

Current research has established obesity as one of the main modifiable risk factors for cognitive impairment. However, evidence on the relationships of total and regional body composition measures as well as sarcopenia with cognitive functioning in the older population remains inconsistent.

**Methods:**

Data are based on 1,570 participants from the British Regional Heart Study (BRHS), a cohort of older British men from 24 British towns initiated in 1978–80, who were re-examined in 2010–12, aged 71–92 years. Cognitive functioning was assessed with the Test-Your-Memory cognitive screening tool. Body composition characteristics assessed using bioelectrical impedance analysis included total fat mass (FM), central FM, peripheral FM, and visceral fat level. Sarcopenia was defined using the European Working Group on Sarcopenia in Older People (EWGSOP) definition of severe sarcopenia and the Foundation for the National Institutes of Health (FNIH) sarcopenia project criteria.

**Results:**

Among 1,570 men, 636 (41 %) were classified in the mild cognitive impairment (MCI) and 133 (8 %) in the severe cognitive impairment (SCI) groups. Age-adjusted multinomial logistic regressions showed that compared with participants in the normal cognitive ageing group, those with SCI were more likely to have waist circumference >102 cm, BMI >30 kg/m^2^, to be in the upper quintile of total FM, central FM, peripheral FM and visceral fat level and to be sarcopenic. The relationships remained significant for total FM (RR = 2.16, 95 % CI 1.29–3.63), central FM (RR = 1.85, 95 % CI 1.09–3.14), peripheral FM (RR = 2.67, 95 % CI 1.59–4.48), visceral fat level (RR = 2.28, 95 % CI 1.32–3.94), BMI (RR = 2.25, 95 % CI 1.36–3.72) and waist circumference (RR = 1.63, 95 % CI 1.05–2.55) after adjustments for alcohol, smoking, social class, physical activity and history of cardiovascular diseases or diabetes. After further adjustments for interleukin-6 and insulin resistance, central FM, waist circumference and sarcopenia were no longer significantly associated with SCI.

**Conclusions:**

Increased levels of peripheral FM, visceral fat level, and BMI are associated with SCI among older people. Distinct pathophysiological mechanisms link regional adipose tissue deposition and cognitive functioning.

## Background

Increased prevalence rates of severe cognitive impairment and dementia are a worldwide health concern because they have been associated with increased mortality rates and posit a global financial burden [1]. Current global estimates suggest that over 35 million people are affected by dementia; with population ageing, the number of sufferers worldwide is expected to increase dramatically by 2050 [1]. Prevalence estimates of mild cognitive impairment (MCI) were shown to range between 2 % to 56 % [2]. Among British men alone, prevalence rates of dementia as high as 14.6 % for those aged 80–84 years were reported in a recent study [3].

Current recommendations suggest that up to one third of dementia cases may be preventable with attention to modifiable risk factors [4]. One of the most consistently proposed risk factors for mid-and late-life cognitive impairment is obesity [4]. In studies assessing the relationship between obesity and cognitive impairment, obesity is commonly measured as body mass index (BMI), waist circumference (WC) or waist-hip ratio (WHR) [5–11]. These proxies of adiposity, particularly BMI, have been criticised as being indices of excess weight rather than body fat or adiposity [12], thus obscuring the underlying role of adipose tissue in cognitive impairment. Other studies have therefore used refined adiposity measures of body composition assessed by means of bioelectrical impedance vector analysis (BIA) or dual energy x-ray absorptiometry (DEXA); the body composition measures most commonly assessed using these techniques were fat mass (FM) and fat-free mass [10, 13–15]. Complete consensus concerning the associations between these adiposity measures and cognitive impairment has not been reached. Specifically some studies have shown a significant positive association between loss of lean mass [11, 16] or increases in FM [16] with impaired cognitive functioning, while a lack of significant associations between these measures has also been documented [11, 14]. Regional deposition of adipose tissue has also been examined in relation to cognitive impairment [13, 15, 17, 18]. Computed tomography (CT)- assessed visceral and abdominal fat were found to be significantly associated with cognitive impairment [17, 18].

The relationship between sarcopenia and cognitive impairment has also been characterised by conflicting evidence. The term sarcopenia was originally used to describe declines in skeletal muscle mass [19], and was shown to be significantly associated with cognitive impairment [10]. More recently, measures of muscle function were added to the operational definitions of sarcopenia [20, 21]. A recent study by Hsu et al. [22] used the definition of The European Working Group on Sarcopenia in Older People (EWGSOP) [20], to define sarcopenia using impaired grip strength and/or walking speed in addition to muscle mass loss [22] and reported positive associations between sarcopenia and cognitive impairment. However, these findings are challenged by findings from the Epidemiologie de l’Osteoporose (EPIDOS) cohort which suggest that sarcopenia is not associated with cognitive impairment when adjusting for potential confounders [23]. Specifically, van Kan et al. [14] compared multiple definitions of sarcopenia, including a) using only muscle mass measurements; b) definitions of sarcopenia which take into account grip strength in addition to muscle mass loss; and c) the EWGSOP definition of sarcopenia. The results suggest that regardless of the definition employed, sarcopenia is not significantly associated with cognitive impairment after adjustments for age, education, disability, physical activity and recruitment centres [23]. More recently, the Foundation for the National Institutes of Health (FNIH) Sarcopenia Project introduced novel criteria for sarcopenia [21]. The potential advantages of the FNIH sarcopenia criteria are a) the two functional components of sarcopenia, i.e. muscle mass and muscle strength, are separate, consistent with the definition of severe sarcopenia according to EWGSOP criteria [20]; and b) low cut-offs for grip strength and muscle mass are used, hence they are less inclusive and can therefore become clinically more relevant [24]. Sarcopenia rates using the definition proposed by the FNIH are yet to be examined in relation to cognitive functioning in the older population.

Due to the inconsistent findings of previous research, in this study we aimed to examine the associations of anthropometric characteristics, regional and total body composition measures assessed using BIA, and functional sarcopenia using both the EWGSOP and FNIH definitions, with cognitive impairment in a large representative sample of the older British men. We hypothesized that total and regional deposits of FM, anthropometric characteristics as well as FNIH-defined sarcopenia will be significantly associated with mild and more severe cognitive impairment.

## Methods

### Study population

Study participants were members of the British Regional Heart Study (BRHS), a study comprising a socially and geographically representative sample of 7735 men aged 40–59 between 1978 and 1980 [25]. Participants were recruited from general practices in 24 towns representative of all major British regions. In 2010–12, 1,570 surviving men were assessed with the Test Your Memory (TYM) during the 30-year re-examination, which was attended by 1722 BRHS participants (55 % response rate). Study members with complete data on the TYM were on average two years younger (M = 78.25, SD = 4.55) in comparison to the remaining ones attending the physical examination (M = 80.2, SD = 4.55; *p* < 0.01), however they did not differ with respect to their social class (*p* = 0.38), education (*p* = 0.29), alcohol consumption (*p* = 0.46), mean BMI (*p* = 0.11) or smoking status (*p* = 0.84). Ethical approval was provided by the National Research Ethics Service (NRES) Committee for London. All men provided written informed consent to the investigations, which were carried out in accordance with the Declaration of Helsinki.

### Outcome measure- cognitive function

Cognitive function was assessed using the TYM [26], a simple 10-task self-assessment cognitive screening instrument which has sound psychometric properties [26, 27], remarkable cross-cultural validity [28–30], and good concurrent validity with established tests [26, 31, 32]. Participants were classified in normal cognitive ageing (NCA), mild cognitive impairment (MCI) or severe cognitive impairment (SCI) groups. Total scores below 33 and scores between 33 and 45 (if older than 80 years of age) or 46 (if younger than 80 years of age) were considered to be indicative of SCI and MCI, respectively, consistent with the scores reported in the original study on TYM by Brown et al. (2009) for patients with Alzheimer’s disease and healthy controls [26], and with the scoring sheet for the TYM available by the Royal College of Psychiatrists [33].

### Anthropometric and body composition charateristics

The anthropometric characteristics considered for this study included *WC*, *WHR*, *BMI* and *mid*-*arm muscle circumference* (*MAMC*)*.* Waist and hip circumference (cm) was measured in duplicate with an insertion tape (CMS Ltd London). Hip circumference was measured at the point of maximum circumference over the buttocks. WC was measured from the midpoint between the iliac crest and the lower ribs measured at the sides. WHR was calculated as WC/(hip circumference). BMI was calculated as weight/(height)^2^ (kg/m^2^). MAMC was calculated as (mid-upper arm circumference) − 0.3142 × (triceps skinfold thickness) [34].

BIA-assessed body composition characteristics were obtained using the TANITA MA-418-BC impedance analyser (Tanita Inc., Tokyo, Japan). The TANITA analyser calculates body fat based on the voltage drop from foot to foot after a small alternating current is applied through contact with two metal foot plates [35]. Assessments included FM (kg), peripheral FM (kg), central FM (kg), visceral fat level (FL) and predicted muscle mass (MM) (kg). Peripheral FM and appendicular MM were defined as the sum of the FM or MM of all four limbs, respectively. Central FM was defined as trunk FM. Visceral FL was based on abdominal BIA [36]. It ranged from 1 to 59 with higher values indicating greater levels of visceral fat. Body composition assessmentes were adjusted for height (m), by dividing by height-squared. BIA has been shown to be influenced by the variability in fat-free mass (FFM) hydration [37, 38], however studies in selected populations have shown that BIA assessments are comparable to the ones obtained by DXA [39, 40] and that it is one of the most simple and efficient ways to obtain body-composition characteristics, particularly in cohort samples where cost-effectiveness is a major issue due to the restricted amount of resources available [41]. The EWGSOP has also approved BIA measurements as a portable alternative to dual-energy X-ray absorptiometry [20].

### Muscle function and sarcopenia

*Gait speed* was assessed by means of a walking test (time taken, in seconds, to walk 3 m at normal walking pace). *Grip strength* (in kilograms) was measured with a Jamar Hydraulic Hand Dynamometer. Three measurements were taken for each hand, and the best of six readings was used for the analysis.

Two definitions of s*arcopenia* were used. The first derived from the EWGSOP definition for severe sarcopenia and included concurrent presence of low lean mass (height-adjusted appendicular muscle mass < =7.23), low grip strength (<30 kg) and low gait speed (<=0.8 m/s) [20]. The second was developed by the FNIH sarcopenia project and included BMI-adjusted low lean mass (<0.789), low grip strenth (grip strength < 26 kg) and slowness (gait speed < =0.8 m/s) [42].

### Covariates

*Insulin-resistance (IR)* was assessed using the Homeostasis Model Assessment (HOMA) as the product of fasting glucose (mmol/l) and insulin (μU/ml) divided by the constant 22.5 [43]. Inflammation was assessed using two markers, *interleukin-6 (IL-6)* (pg/mL) and *C-reactive protein (CRP)* (mg/L). History of cardiovascular diseases (CVD) or diabetes was based on self-report of a doctor diagnosis of coronary thrombosis, myocardial infarction, stroke or diabetes. *Socioeconomic position* was defined based on the longest-held occupation of subjects at study entry (aged 40–59 years) in accordance to the Registrar Generals’ Social Class Classification and has been described elsewhere [44]. *Education* was measured using information asked in a questionnaire in 1996 on the age study members left their full-time education and were classified into three categories using < =14 and < =18 years as the cut-offs. *Physical activity* assessments included walking, cycling and other sporting activities. Physical activity scores were assigned on the basis of frequency and type of activity and the men were divided into 6 groups: none, occasional, light, moderate, moderately-vigorous and vigorous. Subjects were also asked detailed questions about their *smoking status* and *alcohol consumption* habits. Men were classified in four groups according to their smoking habits as current smokers, long term ex-smokers (gave up smoking before 1983), recent ex-smokers and those who never smoked. Alcohol consumption was recorded using questions on the frequency, quantity and type. Heavy drinking was defined as drinking more than six units (1 UK unit = 10 g) of alcohol daily or on most days.

### Statistical analysis

Age-adjusted multinomial logistic regressions were performed to examine the associations between anthropometric and body composition characteristics, muscle function, sarcopenia rates and muscle function with TYM-defined cognitive functioning categories. In all models, the NCA group was used as the reference group. Next, age-adjusted multinomial logistic regressions were performed to examine the relationship between upper tertiles of IR, IL-6 and CRP with cognitive functioning groups. These models were then further adjusted for alcohol consumption, physical activity, social class, smoking and history of CVD and diabetes. Age-adjusted partial correlations were also computed to examine the relationships between anthropometric and body composition characteristics with IR and the inflammatory markers. After the examination of these relationships, we performed further multinomial logistic regressions to assess the associations between those anthropometric and body composition characteristics that were significantly associated with cognitive group membership in the age-adjusted regression models, upon further adjustments for IR, CRP or IL-6, alcohol consumption, physical activity, social class, smoking and history of CVD and diabetes. IR, CRP and IL-6 were natural log-transformed to account for their skewed distribution and were entered in the models as continuous variables. The remaining covariates were entered as categorical variables in the respective models. All analyses were carried out using Stata/IC 13.1 (StataCorp LP, College Station, TX, USA).

## Results

Table 1 summarizes the baseline demographic and lifestyle characteristic of the sample. Among 1,570 men in the study, 801 (51 %) were classified in the NCA, 636 (41 %) in the MCI and 133 (8 %) in the SCI group. Mean age and the proportion (%) of study members reporting moderate to heavy alcohol consumption did not differ between groups, however significantly higher rates of participants with severe cognitive impairments were in a manual social class, left their full-time education when aged < = 14 years, reported being physical inactive and having been smokers (p values < =0.001).

**Table 1:** Baseline characteristics of sample across cognitive function groups in a population-based study of 1570 older British men aged 71–92 years in 2010-12

	Normal cognitive ageing (*n* = 801, 51 %)	Mild cognitive impairment (*n* = 636, 41 %)	Severe cognitive impairments (*n* = 133, 8 %)	*P* value^*^
Total TYM Score (M ± SD)	47.73 ± 1.49	41.21 ± 3.13	27.43 (5.33)	<.001
Age (M ± SD)	78.09 ± 4.39	78.32 ± 4.69	78.88 ± 4.76	0.16
Manual social class, n(%)	272 (35 %)	334 (54 %)	100 (76 %)	<.001
Education, n(%) left full-time education at age 14 years or earlier	138 (18 %)	140 (25 %)	44 (39 %)	<.001
Physical Activity, Inactive, n(%)	274 (36 %)	247 (42 %)	61 (50 %)	0.001
Smoking, Never smoked, n(%)	344 (43 %)	209 (33 %)	41 (31 %)	<.001
Alcohol Consumption, Moderate/Heavy Drinker, n(%)	25 (2 %)	10 (2 %)	3 (2 %)	0.99

^*^p value of respective Chi-square or ANOVA test

Table 2 summarizes the age-adjusted relative risk ratios (RR) of the anthropometric characteristics and the BIA-assessed body composition measures with the TYM-defined cognitive functioning groups. With respect to the anthropometric characteristics, in comparison to participants in the NCA category those classified as having SCI were significantly more likely to have WC > 102 cm (RR = 1.98, 95 % CI 1.36–2.87), BMI > 30 kg/m^2^ (RR = 2.59, 95 % CI 1.72–3.91) and less likely to be in the bottom quintile of MAMC (<=22.09 cm) (RR = 0.57, 95 % CI 0.34–0.97). Participants with SCI were also significantly more likely to be in the upper quintile of central FM (RR = 2.24, 95 % CI 1.45–3.47), peripheral FM (RR = 2.93, 95 % CI 1.90–4.50), visceral FL (RR = 2.77, 95 % CI 1.76–4.36), and overall FM (RR = 2.62, 95 % CI 1.71–4.04) in comparison to the NCA group. Study members classified in the MCI group were also significantly more likely to be in the upper quintile of visceral FM (RR = 1.45, 95 % CI 1.08–1.95) but did not differ compared with those in the NCA group with respect to any of the remaining anthropometric or body composition characteristics.

**Table 2:** Relationships between obesity and regional adipose deposition with cognitive impairment in a study of British men aged 71–92 years in 2010–12

	Normal cognitive ageing (*n* = 801, 51 %)	Mild cognitive impairment (*n* = 636, 41 %)	Severe cognitive impairment (*n* = 133, 8 %)	P value^*^
Anthropometric characteristics				
Waist Circumference				
High Waist Circumference (>102 cm), n(%)	288 (36 %)	259 (41 %)	69 (52 %)	.001
Relative Risk Ratio (95 % CI)	1.00	1.23 (1.00–1.53)	1.98 (1.36–2.87)^‡^	
Waist-Hip Ratio				
Top Quintile (> = 1.00), n(%)	151 (19 %)	126 (21 %)	27 (22 %)	.75
Relative Risk Ratio (95 % CI)	1.00	1.09 (0.83–1.42)	1.19 (0.75–1.89)	
MAMC				
Bottom Quintile (<=22.09 cm), n(%)	169 (21 %)	125 (20 %)	19 (14 %)	0.20
Relative Risk Ratio (95 % CI)	1.00	0.89 (0.69–1.16)	0.57 (0.34–0.97)^†^	
BMI				
Obese (>30 kg/m^2^), n(%)	137 (17 %)	124 (20 %)	44 (34 %)	<.001
Relative Risk Ratio (95 % CI)	1.00	1.19 (0.91–1.57)	2.59 (1.72–3.91)^‡^	
BIA-assessed regional and total adiposity measures				
Central FM				
Top Quintile (> = 6.3 kg/m^2^), n(%)	135 (18 %)	112 (20 %)	37 (33 %)	.001
Relative Risk Ratio (95 % CI)	1.00	1.12 (0.84–1.48)	2.24 (1.45–3.47)^‡^	
Peripheral FM				
Top Quintile (> = 3.4 kg/m^2^), n(%)	132 (18 %)	110 (19 %)	42 (38 %)	<.001
Relative Risk Ratio (95 % CI)	1.00	1.13 (0.85–1.50)	2.93 (1.90–4.50)^‡^	
Visceral fat level				
Top Quintile (> = 6.9), n(%)	109 (16 %)	114 (22 %)	37 (35 %)	<.001
Relative Risk Ratio (95 % CI)	1.00	1.45 (1.08–1.95)^†^	2.77 (1.76–4.36)^‡^	
Total FM				
Top Quintile (> = 9.7 kg/m^2^), n(%)	131 (18 %)	113 (20 %)	40 (35 %)	<.001
Relative Risk Ratio (95 % CI)	1.00	1.18 (0.89–1.55)	2.62 (1.71–4.04)^‡^	

*MAMC* Mid-arm muscle circumference, *BMI* Body-Mass Index, *FM* Fat Mass

^*^ *p*-value of Chi-Square test

^†^ *p* < .05

^‡^ *p* < .01

risk ratios are age-adjusted

Table 3 presents the relationships between sarcopenia rates and cognitive functioning. In total, 2.5 % (*n* = 34) of study members were identified as sarcopenic using the EWGSOP-definition of severe sarcopenia and 2.7 % (*n* = 36) using the one proposed by the FNIH sarcopenia project. Higher rates of participants classified as sarcopenic were observed among the group of participants with SCI in comparison to those in the NCA group for both, the EWGSOP (RR = 4.65, 95 % CI 1.78–12.19) and the FNIH definition (RR = 2.90, 95 % CI 1.04–8.05).

**Table 3:** Relationships between EWGSOP and FNIH-defined sarcopenia with cognitive impairment in a study of British men aged 71–92 years in 2010-12

	Normal cognitive ageing (*n* = 801, 51 %)	Mild cognitive impairment (*n* = 636, 41 %)	Severe cognitive impairment (*n* = 133, 8 %)	*P* value^*^
Lean mass, grip strength and gait speed cut-offs				
Appendicular Muscle Mass/BMI- *FNIH* cut-off (<0.789), n(%)	119 (16 %)	131 (23 %)	35 (32 %)	<.001
Relative Risk Ratio (95 % CI)	1.00	1.57 (1.19–2.09)^‡^	2.39 (1.51–3.77)^‡^	
Appendicular Muscle Mass/height- *EWGSOP* cut-off (<=7.23), n(%)	185 (25 %)	137 (24 %)	31 (19 %)	.37
Relative Risk Ratio (95 % CI)	1.00	0.95 (0.73–1.23)	0.65 (0.39–1.08)	
Grip strength- *FNIH* cut-off (<26 kg), n(%)	190 (25 %)	169 (28 %)	38 (29 %)	.30
Relative Risk Ratio (95 % CI)	1.00	1.16 (0.90–1.48)	1.20 (0.79–1.83)	
Grip strength- *EWGSOP* cut-off (<30 kg), n(%)	257 (33 %)	234 (38 %)	57 (44 %)	.02
Relative Risk Ratio (95 % CI)	1.00	1.23 (0.98–1.54)	1.48 (1.01–2.19)^†^	
Gait Speed (<=0.8 m/s), n(%)	187 (24 %)	223 (36 %)	57 (45 %)	<.001
Relative Risk Ratio (95 % CI)	1.00	1.83 (1.45–2.33)^‡^	2.60 (1.74–3.87)^‡^	
Sarcopenia				
Severe Sarcopenia- *EWGSOP* definition, n(%)	11 (1.5 %)	15 (2.8 %)	8 (7.5 %)	.001
Relative Risk Ratio (95 % CI)	1.00	1.78 (0.80–3.95)	4.65 (1.78–12.19)^‡^	
Sarcopenia- *FNIH* definition, n(%)	13 (1.8 %)	17 (3.2 %)	6 (5.7 %)	.04
Relative Risk Ratio (95 % CI)	1.00	1.70 (0.81–3.57)	2.90 (1.04–8.05)^†^	

*EWGSOP* European Working Group on Sarcopenia in Older People, *FNIH* Foundation for the National Institutes of Health Sarcopenia Project

^*^p-value of respective Chi-Square test

^†^ *p* < .05

^‡^ *p* < .01

risk ratios are age-adjusted

Table 4 summarizes the relationships between the three cognitive functioning groups with IR, IL-6 and CRP levels. The results suggest that participants in the upper tertile of IR (RR = 1.87, 95 % CI 1.25–2.80), IL-6 (RR = 2.03, 95 % CI 1.37–3.01), and CRP (RR = 1.55, 95 % CI 1.05–2.30) were significantly more likely to be in the SCI group in comparison to the NCA group after adjusting for age. Upon further adjustments for alcohol consumption, social class, physical activity, smoking and history of CVDs or diabetes, only the relationship between IL-6 and SCI retained its significance (RR = 1.90, 95 % CI 1.20–3.01) and therefore it was considered as a covariate in subsequent regression models.

**Table 4:** Relationships between insulin-resistance and inflammatory markers with cognitive impairment

	Normal cognitive ageing (*n* = 801, 51 %)	Mild cognitive impairment (*n* = 636, 41 %)	Severe cognitive impairment (*n* = 133, 8 %)	*P* value^*^
HOMA				
Top Tertile (> = 2.63), n(%)	221 (32 %)	186 (33 %)	53 (46 %)	.01
Relative Risk Ratio (95 % CI)^a^	1.00	1.05 (0.83–1.33)	1.87 (1.25–2.80)^ǁ^	
Relative Risk Ratio (95 % CI)^b^	1.00	0.92 (0.69–1.22)	1.58 (0.97–2.58)	
Interleukin-6				
Top Tertile (> = 3.8 pg/L), n(%)	223 (30 %)	211 (35 %)	58 (48 %)	<.001
Relative Risk Ratio (95 % CI)^a^	1.00	1.26 (1.00–1.59)	2.03 (1.37–3.01)^ǁ^	
Relative Risk Ratio (95 % CI)^b^	1.00	1.03 (0.79–1.35)	1.90 (1.20–3.01)^ǁ^	
C-Reactive Protein				
Top Tertile (> = 2.2 mg/L), n(%)	234 (31 %)	202 (34 %)	52 (43 %)	.05
Relative Risk Ratio (95 % CI)^a^	1.00	1.10 (0.88–1.39)	1.55 (1.05–2.30)^§^	
Relative Risk Ratio (95 % CI)^b^	1.00	1.00 (0.77–1.30)	1.40 (0.89–2.21)	

HOMA: Homeostasis Model Assessment for Insulin resistance

^*^p-value of respective Chi-square test

^§^ *p* < .05

^ǁ^ *p* < .01

^a^Models adjusted for age

^b^Models adjusted for age, alcohol consumption, social class, physical activity, smoking, history of CVDs and diabetes

Table 5 presents the correlation coefficients for the relationships between anthropometric and body composition characteristics with IR and the inflammatory markers. With the exception of the relationship between CRP and MAMC (r = .04, *p* = 0.11) all other age-adjusted partial correlation coefficients were statistically significant. These ranged from 0.12 (r = 0.12, *p* < 0.001) for the relationship between MAMC and IR, to 0.48 for the relationship between total FM and IR (r = 0.48, *p* < 0.001). Results of age-adjusted logistic regression model showed additionally that study members classified as having sarcopenia using the FNIH definition were more likely to be in the upper tertile of IR (odds ratio (OR) = 2.27, 95 % CI 1.10–4.68), CRP (OR = 3.03, 95 % CI 1.44–6.35) and IL-6 (OR = 2.97, 95 % CI 1.39–6.32). Those classified as having sarcopenia using the EWGSOP definition of severe sarcopenia did not differ significantly with respect to any of the metabolic or inflammatory markers in comparison to non-sarcopenic study participants.

**Table 5:** Age-adjusted correlations between adiposity measures, insulin resistance and inflammatory markers

	Insulin resistance	Interleukin-6	C-reactive protein
Waist Circumference	.45^†^	.19^†^	.23^†^
Waist-Hip Ratio	.33^†^	.17^†^	.22^†^
MAMC	.12^†^	.12^†^	.04
BMI	.43^†^	.18^†^	.20^†^
Central Fat Mass	.46^†^	.19^†^	.23^†^
Peripheral Fat Mass	.47^†^	.16^†^	.22^†^
Visceral Fat Level	.41^†^	.18^†^	.22^†^
Total Fat Mass	.48^†^	.18^†^	.23^†^

All partial correlations are age-adjusted

*MAMC* Mid-arm muscle circumference, *BMI* Body-Mass Index

^*^ *p* < .05

^†^ *p* < .01

As shown in Table 6, after further adjusting for alcohol, smoking, social class, physical activity, history of CVD, and history of diabetes, study members with WC > 102 cm (RR = 1.63, 95 % CI 1.05–2.55), BMI > 30 kg/m^2^(RR = 2.25, 95 % CI 1.36–3.72), those in the upper quintile of central FM (RR = 1.85, 95 % CI 1.09–3.14), peripheral FM (RR = 2.67, 95 % CI 1.59–4.48), visceral FM (RR = 2.28, 95 % CI 1.32–3.94) and total FM (RR = 2.16, 95 % CI 1.29–3.63) were still more likely to be in the SCI in comparison to the NCA group. With the exception of central FM and WC these relationships remained significant after adjustments for IL-6; additionally, being in the upper quintile of overall FM or central FM, or having WC > 102 cm, was no longer significantly associated with membership in the SCI group (all p values > 0.05) after adjustments for IR. Finally, sarcopenia using either of the two definitions employed was not significantly associated with cognitive impairment after adjustments for alcohol, smoking, social class, physical activity, history of CVD, history of diabetes, IL-6 and IR. The results remained consistent when adjusting for CRP as an additional measure of inflammation and after substituting social class in the regression models with education. The latter ones were not controlled for in the same regression models because they were highly correlated and would therefore result in inflated estimates of the regression coefficients.

**Table 6:** Associations between adiposity measures, sarcopenia and cognitive function after adjustments for sociodemographic characteristics, lifestyle factors and inflammatory or metabolic markers in a study of British men aged 71–92 years in 2010-12

	Mild cognitive impairment (*n* = 636, 41 %)			Severe cognitive impairment (*n* = 133, 8 %)		
	RR (95 % CI)^a^	RR (95 % CI)^b^	RR (95 % CI)^c^	RR (95 % CI) ^a^	RR (95 % CI) ^b^	RR (95 % CI)^c^
Waist Circumference (>102 cm)	1.14 (0.88–1.46)	1.25 (0.95–1.66)	1.15 (0.88–1.49)	1.63 (1.05–2.55)^§^	1.55 (0.94–2.55)	1.52 (0.95–2.43)
BMI (>30 kg/m^2^)	1.18 (0.86–1.62)	1.22 (0.85–1.75)	1.16 (0.83–1.62)	2.25 (1.36–3.72)^ǁ^	2.09 (1.17–3.72)^§^	2.30 (1.36–3.91)^ǁ^
MAMC Bottom Quintile (<=22.09 cm)	0.91 (0.67–1.23)	0.87 (0.63–1.20)	0.87 (0.64–1.19)	0.76 (0.42–1.36)	0.69 (0.37–1.31)	0.76 (0.42–1.40)
Central Fat Mass Top Quintile (> = 6.3 kg/m^2^)	0.99 (0.71–1.37)	1.20 (0.83–1.74)	1.06 (0.75–1.49)	1.85 (1.09–3.14)^§^	1.36 (0.72–2.59)	1.65 (0.93–2.91)
Peripheral Fat Mass Top Quintile (> = 3.4 kg/m^2^)	1.00 (0.72–1.40)	1.16 (0.80–1.69)	1.06 (0.75–1.50)	2.67 (1.59–4.48)^ǁ^	2.25 (1.23–4.13)^ǁ^	2.57 (1.48–4.45)^ǁ^
Visceral Fat Level Top Quintile (> = 6.9/m^2^)	1.33 (0.94–1.88)	1.54 (1.05–2.28)^§^	1.35 (0.94–1.95)	2.28 (1.32–3.94)^ǁ^	1.99 (1.05–3.78)^§^	2.27 (1.27–4.06)^ǁ^
Total Fat Mass Upper Quintile (> = 9.7 kg/m^2^)	1.04 (0.75–1.44)	1.28 (0.88–1.85)	1.13 (0.80–1.59)	2.16 (1.29–3.63)^ǁ^	1.70 (0.92–3.15)	2.01 (1.16–3.49)^§^
Sarcopenia (Severe) - *EWGSOP* definition	1.31 (0.53–3.22)	1.41 (0.56–3.54)	1.40 (0.56–3.51)	2.79 (0.89–8.74)	2.67 (0.78–9.20)	2.84 (0.89–9.09)
Sarcopenia *- FNIH* definition	1.41 (0.58–3.39)	1.81 (0.71–4.64)	1.72 (0.67–4.40)	1.30 (0.32–5.18)	1.64 (0.39–6.91)	1.47 (0.35–6.12)

Reference is the normal cognitive ageing group

*RR* Relative risk ratios, *MAMC* Mid-arm muscle circumference, *BMI* Body-Mass Index, *CVD* Cardiovascular Disease, *IR* Insulin-resistance, *IL-6* Interleukin-6, *FNIH* Foundation for the National Institutes of Health, *EWGSOP* European Working Group on Sarcopenia in Older People

^§^ *p* < .05

^ǁ^ *p* < .01

^a ^Models adjusted for age, alcohol consumption, smoking, social class, physical activity, history of CVD, and history of diabetes

^b^ Models adjusted for age, alcohol consumption, smoking, social class, physical activity, history of CVD, history of diabetes and IR

^c^ Models adjusted for age, alcohol consumption, smoking, social class, physical activity, history of CVD, history of diabetes and IL-6

## Discussion

The findings of this study suggest that cognitive functioning is associated not only with conventional anthropometric measures of total adiposity, particularly BMI and WC, but also with refined BIA-assessed measures of total and regionally deposited FM. The relationships between BMI, peripheral FM, visceral FM and SCI remained significant upon adjustments for lifestyle and sociodemographic factors, as well as inflammatory and metabolic markers. In contrast, central FM and WC did not retain their significant associations with SCI upon adjustments for IR or IL-6, suggesting unique pathophysiological pathways linking regional FM deposition and cognitive functioning. Higher rates of participants with excess visceral FM were also identified among people with MCI. Finally, although higher rates of individuals classified as sarcopenic using the EWGSOP and the FNIH definition were found among the SCI compared to the NCA group, these relationships did not retain their significance level after adjustments for relevant covariates.

### Comparison with other studies

The results of this study are comparable with the majority of studies examining the relationships between commonly assessed markers of overall adiposity, such as BMI or WC, with cognitive abilities which have reported significant positive associations [10, 16, 45]. While a recent large-scale study suggested that obesity acts as a protective factor for dementia, the reasons of these associations are unclear [46]. Moreover, in agreement with a study using BIA-assessments of lean mass and FM [16], our study identified significant associations between several refined FM measures and cognitive dysfunctions.

However, while the effects of BMI and visceral fat remained significant after adjustments for inflammatory and metabolic markers, WC and central FM were not significantly associated with SCI after adjustments for inflammatory markers or IR. WC appears, therefore, to be an index of central adiposity while BMI rather a measure of excess weight rather than body fat, in agreement with previous recommendations [47]. These findings are also consistent with current evidence suggesting that the negative effects of visceral adiposity on brain volume is reduced yet persists after controlling for IR [15], and that central adiposity plays a role in inflammatory processes which appears to be independent of total body FM [48].

In line with findings of a recent study on 731 elderly subjects which did not identify a significant link between sarcopenia and global cognitive functioning scores, but rather just the verbal fluency cognitive domain [49], our results suggest that sarcopenia is not significantly associated with cognitive functioning groups defined using total TYM scores. Moreover, available evidence suggests that impaired cognitive functions and EWGSOP-defined sarcopenia are significantly associated [22], although these relationships have been accounted for by confounding variables in a study by van Kan et al. [14, 23]. Our study confirms this finding and extends it using the FNIH definition of sarcopenia, in addition to the EWGSOP definition of severe sarcopenia. While age-adjusted regression models suggested that sarcopenia using is more prevalent among people with SCI, these differences were accounted for by alcohol consumption, smoking, physical activity, history of CVD or diabetes and metabolic and inflammatory markers. Interestingly, of the three components comprising the definitions of sarcopenia, i.e. low lean mass, slowness and low grip strength, the latter showed the strongest association with SCI. This finding is likely to reflect a reverse causation bias according to findings based on prospectively collected data which suggest that cognitive decline precedes the onset of muscle weakness [50]. The temporal relationship between the newer definitions of sarcopenia and cognitive impairment should therefore be replicated using longitudinal data.

### Suggested pathophysiological pathways linking adiposity and cognitive functions

One suggested physiological pathway linking adipose tissue deposition and severe cognitive impairment is through alterations in IR [51, 52]. Specifically, adipocytes have a metabolically active role on the central nervous system by modulating biological pathways, including impairing insulin sensitivity [53, 54]. Impaired brain insulin signalling has been linked to AD [55], with increased IR being additionally demonstrated to be associated with synaptic failure, brain atrophy, and cognitive declines [55]. Furthermore, it has been shown that trunk FM is unfavorably associated with IR [56]. Since the results of this study show that the relationships between central FM and WC were no longer significant with AD range of cognitive functioning after controlling for IR, it is possible that the relationship between central adipose tissue deposition and cognitive functioning is, at least partially, mediated by IR. The effects of peripheral FM pertained after adjusting for IR and metabolic markers, suggesting a potentially more complex pathophysiological mechanism linking peripheral FM and cognitive impairment.

Another suggested pathway relating excess adipose tissue deposition and cognitive impairment is via inflammatory processes. In our study, the associations between SCI and WC or central FM were not significant after adjustments for IL-6 or CRP. Research suggests that central adiposity plays a role in inflammatory processes which appears to be independent of total body fat mass [48], and the defective ability to maintain low inflammation levels contributes in its turn to the onset of AD [57]. It is therefore possible that differing regional depositions of adipose tissue follow a different physiological pathway to affect cognitive functions; however, these are yet to be systematically tested, particularly in studies making use of prospective data.

### Strengths and limitations

We believe that this is the first study examining relative adiposity measures, detailed measures of FM, and severe sarcopenia defined using the EWGSOP and the FNIH operational definitions in relation to both, mild and severe cognitive impairment in a general-population sample of older people. The BRHS is a highly representative sample of the older male UK population which has been successful in keeping attrition rates at very low levels (98 % follow-up rate).

One of the limitation of this study relates to the self-report nature of the TYM. However, we believe that it is an accurate proxy of cognitive function because in clinical settings it has shown good concurrent validity with established tests [26, 31, 32]. We have also shown in a recent study that the cardiometabolic and sociodemographic correlates of TYM-defined cognitive groups in the BRHS are identical to those extracted using established screening tools [58]. In addition, the proportion of participants classified as being in the MCI or SCI range of cognitive functioning using the TYM, is in line with the prevalence rates reported in studies using different diagnostic criteria [1, 2]. The available literature has shown that the respective prevalence rates for mild forms of cognitive dysfunction and Alzheimer’s Disease range between 2 % to 56 % [2] and 2 % to 8.5 %, respectively, for those aged 60 years and over [1].

However, the cross-sectional nature of this investigation means that the direction of causation in the associations observed cannot be directly inferred. To address this, we performed exploratory analyses comparing BMI assessments in our study population measured 10 years prior to administration of the TYM. Results of these analyses showed that participants classified in the SCI group had significantly increased BMI 10 years prior to their cognitive screening. It is, therefore, more likely that increased obesity precedes cognitive impairment rather than vice versa. The results presented in this study are also subject to the sampling bias introduced by the 55 % overall response rate of the BRHS. Nevertheless, study members who were lost between the 20- and the 30-year re-examination were more likely to have a higher waist circumference, insulin resistance and IL-6 levels compared with those with complete data in both assessments; therefore, it is likely that the observed association between cognitive impairment and waist circumference would be more prominent if response rates were higher. In contrast, mean BMI rates did not differ significantly between the groups. Results relative to sarcopenia rates should be interpreted with caution because these analyses were somewhat underpowered given the low prevalence of sarcopenia. In addition, 73 % of the sample attending the assessment completed the TYM, which poses an additional selection bias. Finally, the BRHS includes only white European men, therefore the generalisability of the findings to women and other ethnic groups is limited.

## Conclusions

The results of this study suggest that BMI, WC, as well as BIA-assessed total FM, peripheral FM and central FM are strongly associated with severe cognitive impairment. Central FM and WC are no longer associated with SCI after adjustuments for metabolic and inflammatory markers suggesting that regional depositions of adipose tissue might affect cognitive abilities through different biological mechanisms. Therefore, specific regional adipose tissue deposition patterns and not just the accumulation of fat mass might be one of the missing links in the attempts to understand the pathophysiological mechanisms linking obesity and cognitive impairment. Finally, sarcopenia, when defined using strict cut offs for lean mass loss, gait speed and grip strenth is not associated with SCI; this relationship appears to be entirely accounted for by age, alcohol consumption, smoking, social class, physical activity, history of CVD, history of diabetes and inflammatory or metabolic markers.

## Abbreviations

BIA: Bioimpedance analysis
CI: Confidence Interval
CT: Computed Tomography
BMI: Body-Mass Index
BRHS: British Regional Heart Study
CRP: C-Reactive Protein
CVD: Cardiovascular Diseases
DEXA: Dual Energy X-ray Absorptiometry
EPIDOS: Epidemiologie de l’Osteoporose study
EWGSOP: European Working Group on Sarcopenia in Older People
FL: Fat Level
FM: Fat mass
FNIH: Foundation for the National Institutes of Health
HOMA: Homeostasis Model Assessment
IL-6: Interleukin-6
IR: Insulin Resistance
MAMC: Mid-Arm Muscle Circumference
MCI: Mild Cognitive Impairment
NCA: Normal Cognitive Ageing
NRES: National Research Ethics Service
OR: Odds Ratio
SCI: Severe Cognitive Impairment
RR: Relative Risk Ratio
TYM: Test Your Memory
WC: Waist Circumference
WHR: Waist-Hip ratio
